# Measuring universal health coverage in reproductive, maternal, newborn and child health: An update of the composite coverage index

**DOI:** 10.1371/journal.pone.0232350

**Published:** 2020-04-29

**Authors:** Fernando C. Wehrmeister, Aluisio J. D. Barros, Ahmad Reza Hosseinpoor, Ties Boerma, Cesar G. Victora

**Affiliations:** 1 International Center for Equity in Health, Post-Graduate Programme in Epidemiology, Federal University of Pelotas, Pelotas, Brazil; 2 Division of Data, Analytics and Delivery, World Health Organization, Geneva, Switzerland; 3 Center for Global Public Health, University of Manitoba, Winnipeg, Canada; Jinan University, CHINA

## Abstract

**Background:**

Monitoring universal health coverage in reproductive, maternal and child health requires appropriate indicators for assessing coverage and equity. In 2008, the composite coverage index (CCI)–a weighted average of eight indicators reflecting family planning, antenatal and delivery care, immunizations and management of childhood illnesses–was proposed. In 2017, the CCI formula was revised to update the family planning and diarrhea management indicators. We explored the implications of adding new indicators to the CCI.

**Methods:**

We analysed nationally representative surveys to investigate how addition of early breastfeeding initiation (EIBF), tetanus toxoid during pregnancy and post-natal care for babies affected CCI levels and the magnitude of wealth-related inequalities. We used Pearson’s correlation coefficient to compare different formulations, and the slope index of inequalities [SII] and concentration index [CIX] to assess absolute and relative inequalities, respectively.

**Results:**

47 national surveys since 2010 had data on the eight variables needed for the original and revised formulations, and on EIBF, tetanus vaccine and postnatal care, related to newborn care. The original CCI showed the highest average value (65.5%), which fell to 56.9% when all 11 indicators were included. Correlation coefficients between pairs of all formulations ranged from 0.93 to 0.99. When analysed separately, 10 indicators showed higher coverage with increasing wealth; the exception was EIBF (SII = -2.1; CIX = -0.5). Inequalities decreased when other indicators were added, especially EIBF–the SII fell from 24.8 pp. to 19.2 pp.; CIX from 7.6 to 6.1. The number of countries with data from two or more surveys since 2010 was 30 for the original and revised formulations and 15 when all the 11 indicators were included.

**Conclusions:**

Given the growing importance of newborn mortality, it would be desirable to include relevant coverage indicators in the CCI, but this would lead a reduction in data availability, and an underestimation of coverage inequalities. We propose that the 2017 version of the revised CCI should continue to be used.

## Introduction

Sustainable Development Goal (SDG) number three aims to ensure healthy lives and promote wellbeing for all at all ages [1]. The target 3.8 is related to the achievement of universal health coverage (UHC) “*including financial risk protection*, *access to quality essential health care services*, *and access to safe*, *effective*, *quality*, *and affordable essential medicine and vaccines for all*” [1, 2]. This target includes two topics: coverage of essential health services and catastrophic spending on health [2]. In the present article we discuss the choice of coverage indicators for women and children to be included in a summary index, based upon indicators of access and utilization of services and interventions.

Because of difficulties in interpreting levels and trends in several coverage indicators across many countries, the Countdown to 2015 (now Countdown to 2030) initiative proposed a summary coverage index for reproductive, maternal, newborn and child health (RMNCH) interventions. The original index, introduced in 2008, was defined as the “coverage gap” [3], expressing how far average levels were from universal coverage. It soon became evident that many policymakers had difficulty in interpreting a measure that was expressed as a gap, rather than actual coverage levels. Thus, the coverage gap was reframed as the “composite coverage index” (CCI) in 2013. [4, 5] The CCI is based upon information derived from national surveys, being a weighted average of coverage levels in four intervention areas with different service delivery strategies: contraception, antenatal and delivery care, child immunization and case management for common illnesses of children. The CCI, being based on eight different coverage indicators, was less affected by sampling variability than standalone indicators. It also proved to be strongly associated with outcomes such as under-five mortality rate and stunting prevalence [6]. Also, analyses of CCI according to wealth, area of residence, or geographical regions within a country provided essential information on which groups of women and children were lagging behind [1, 6, 7], which is relevant to the SDG 17.18 requirement for stratified data analyses [1, 2].

The original CCI was revised in 2017 to take into account changes in some of its component indicators, particularly the restriction of the contraception indicator to modern methods, the increase in the number of recommended antenatal visits, and the replacement of oral rehydration therapy with the use of oral rehydration salts for diarrhoea [7, 8].

Newborn deaths now account for almost half of all deaths of children under the age of five years [9], and reducing newborn mortality is a key SDG target. The “Every Newborn Action Plan”, launched in 2015, proposed a series of coverage indicators relevant to newborn health, in addition to the antenatal and delivery care indicators that are part of the CCI. Based upon a review of indicators that are currently included in national demographic and health surveys, we identified three indicators that are related to newborn survival: tetanus toxoid for mothers during pregnancy, early breastfeeding initiation and postnatal care for babies in the 48 hours after delivery. Early initiation of breastfeeding, along with presence of a skilled attendant at delivery, is one of the core indicators in the newborn action plan [10]. In the present analyses, we examined how the inclusion of newborn coverage indicators in the CCI would affect its calculation, with particular emphasis on data availability and on the ability to expose socioeconomic inequalities.

## Materials and methods

The present analyses were based on nationally representative surveys from low- and middle-income countries (LMICs) carried out since 2010. These included the last Demographic and Health Surveys (DHS) or Multiple Indicator Cluster Surveys (MICS) a country could have, which were harmonized by the International Center for Equity in Health [11]. Details on the analytic approach used for measuring intervention coverage are available elsewhere [4, 11].

The CCI is a weighted average of essential maternal and child health interventions along the continuum of care [3, 4, 6]. The original CCI [3, 6] included the following indicators in four stages of the continuum of care: reproductive health (demand for family planning satisfied with any methods (DFPS)); maternal health (at least one antenatal care visit with a skilled provider (ANC1) and skilled birth attendant (SBA); child immunizations (one dose of bacile Calmette-Guérin (BCG), three doses of diphtheria-pertussis-tetanus (DPT3), and one dose of measles (MSL) vaccines; and management of childhood illnesses (oral rehydration therapy for diarrhoea (ORT), and care-seeking for suspected childhood pneumonia (CAREP)). The four stages had equal weights. In the immunizations component, DPT coverage received twice the weight of other vaccines because there was a need for more than one contact with the provider. The formula of the original CCI is shown in the Table 1.

**Table 1:** Alternative formulations of the composite coverage index (CCI).

Original CCI	$\frac{DFPS+\frac{\left(ANC1+SBA\right)}{2}+\frac{\left(MSL+BCG+2*DPT3\right)}{4}+\frac{\left(ORT+CAREP\right)}{2}}{4}$
Revised CCI	$\frac{DFPSmo+\frac{\left(ANC4+SBA\right)}{2}+\frac{\left(MSL+BCG+2*DPT3\right)}{4}+\frac{\left(ORS+CAREP\right)}{2}}{4}$
Revised CCI + early BF initiation	$\frac{DFPSmo+\frac{\left(ANC4+SBA\right)}{2}+EIBF+\frac{\left(MSL+BCG+2*DPT3\right)}{4}+\frac{\left(ORS+CAREP\right)}{2}}{5}$
Revised CCI + early BF initiation + tetanus toxoid for pregnant women	$\frac{DFPSmo+\frac{\left(ANC4+SBA\right)}{2}+\frac{EIBF+TET}{2}+\frac{\left(MSL+BCG+2*DPT3\right)}{4}+\frac{\left(ORS+CAREP\right)}{2}}{5}$
Revised CCI + early BF initiation + postnatal care for babies	$\frac{DFPSmo+\frac{\left(ANC4+SBA\right)}{2}+\frac{EIBF+PNCALL}{2}+\frac{\left(MSL+BCG+2*DPT3\right)}{4}+\frac{\left(ORS+CAREP\right)}{2}}{5}$
Revised CCI + early BF initiation + tetanus toxoid for pregnant women + postnatal care for babies	$\frac{DFPSmo+\frac{\left(ANC4+SBA\right)}{2}+\frac{EIBF+TET+PNCALL}{3}+\frac{\left(MSL+BCG+2*DPT3\right)}{4}+\frac{\left(ORS+CAREP\right)}{2}}{5}$

ANC1 = at least one antenatal care visit with skilled provider; ANC4 = at least four antenatal care visits, regardless the provider; BCG = Bacillus Calmette-Guerin immunization; DFPS = demand for family planning satisfied; CAREP = careseeking for suspected acute respiratory infection; DFPSmo = demand for Family planning satisfied with modern methods; DPT3 = 3 doses of diphteria-tetanus-pertussis immunization; EBF = early breastfeeding initiation (within 2 hours after delivery); MSL = measles immunization; ORS = oral rehydration salts for diarrhoea; ORT = oral rehydration therapy for diarrhoea with continued feeding practices; PNCALL = postnatal care for babies within 48 hours after delivery; SBA = skilled birth attendance; and TET = 2 doses of tetanus toxoid for mother during pregnancy.

In the 2017 report for the Countdown to 2030 [7, 8], three indicators were replaced to reflect changes in global recommendations: DFPS with DFPSmo (modern methods only), ANC1 by ANC4 (four or more visits) and ORT by ORS (oral rehydration salts). This became known as the revised CCI (see Table 1).

In the present analyses, we explored different options for adding newborn health as a fifth component to the CCI by incorporating the following survey-based indicators: early initiation of breastfeeding (EIBF), tetanus toxoid given during pregnancy (TET) and post-natal care visit by the baby within 48 hours after delivery (PNCALL). This led to three additional formulations for the CCI (see Table 1).

To explore how the different versions of the CCI were able to detect socioeconomic-related inequalities, we used household wealth quintiles based on household assets and characteristics of the dwelling. This index is calculated through principal component analysis. The first quintile represents the poorest fifth whereas the last quintile includes the wealthiest fifth of all households in the survey sample. Details on how the wealth index is estimated can be found elsewhere [12].

We calculated summary statistics, including mean, standard deviation, median, minimum and maximum values for the CCI coverage in the most recent survey carried out in each country with publicly available data. Within each country, summary measures [13, 14] were calculated for absolute (slope index of inequality) and relative (concentration index) inequalities. The slope index is calculated through logistic regression and expresses the difference, in percentage points, between the predicted values at the bottom and the top of the wealth scale. It varies from -100 to +100 percentage points [4, 13, 14]. The concentration index is calculated similarly to the Gini index, by plotting cumulative coverage over wealth quintiles, and how the resulting curve differs from a diagonal line that indicates perfect equality. The concentration index is a dimensionless statistic that ranges from -100 to +100 [4, 13, 14]. For both indices, positive values mean that coverage is higher for the richest, while negative values indicate the opposite. Annex 1 provides details of the calculation of both indices.

Pearson correlation coefficients were used to assess the correlation between different CCI versions. All analyses were performed using Stata version 15.0 (Stata Corp., College Station, United States of America). Ethical clearance was obtained by the national agencies responsible for each survey. All analyses relied on publicly available, anonymized databases.

## Results

We analyzed 60 countries (39 DHS and 21 MICS) with at least one publicly available survey carried out since 2010. Detailed information on these countries and corresponding CCI estimates are shown in S1 Table. For all countries, it was possible to calculate the original CCI, revised CCI and revised CCI plus early breastfeeding initiation. The numbers of countries with data were reduced to 55, 52 and 47 when tetanus vaccine, postnatal care, or both were added to the formulation, respectively (S1 Table).

**Fig 1:**
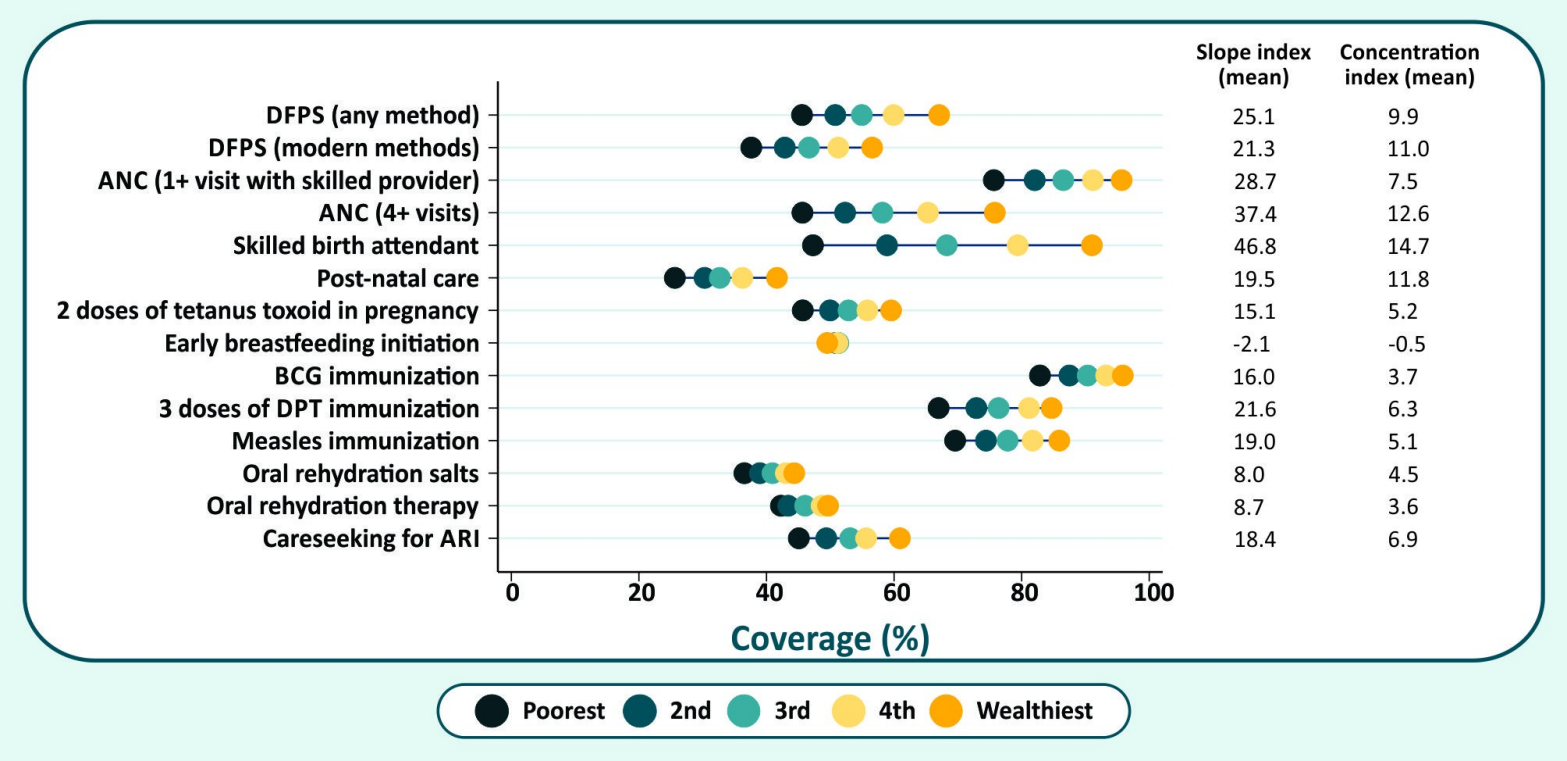
Average coverage levels by wealth quintile of each intervention included in the different CCI formulations.

Table 2 shows descriptive statistics for the CCI formulations in the national samples as well as for the poorest and wealthiest quintiles in the 47 countries for which all CCI versions could be computed, whereas Fig 1 shows the average levels of coverage across the same countries for each of the interventions included in the CCI formulations, by wealth quintile.

**Table 2:** Summary statistics for different formulations of the CCI in 47 countries.

Indicator	Mean	Median	Minimum	Maximum
*National*				
Original CCI	65.5	65.4	33.5	86.6
Revised CCI	59.8	61.5	28.9	81.5
Revised CCI + early BF initiation	58.0	59.5	27.0	76.5
Revised CCI + early BF initiation + post-natal care for babies	56.4	57.8	25.1	79.5
Revised CCI + early BF initiation + tetanus vaccine	57.9	58.5	29.7	77.0
Revised CCI + early BF initiation + tetanus vaccine + post-natal care for babies	56.9	60.2	27.6	79.3
*Poorest quintile (average across countries)*				
Original CCI	55.7	58.0	18.6	83.6
Revised CCI	49.9	50.7	15.4	78.9
Revised CCI + early BF initiation	50.3	52.2	18.0	74.5
Revised CCI + early BF initiation + tetanus vaccine	47.7	49.2	16.3	77.3
Revised CCI + early BF initiation + post-natal care for babies	49.5	53.7	17.2	75.6
Revised CCI + early BF initiation + tetanus vaccine + post-natal care for babies	48.1	51.2	16.3	77.1
*Richest quintile (average across countries)*				
Original CCI	75.9	75.4	50.6	87.3
Revised CCI	70.4	70.2	45.9	83.6
Revised CCI + early BF initiation	66.0	66.4	41.0	80.9
Revised CCI + early BF initiation + tetanus vaccine	65.5	66.7	39.5	81.1
Revised CCI + early BF initiation + post-natal care for babies	66.8	67.2	45.5	81.8
Revised CCI + early BF initiation + tetanus vaccine + post-natal care for babies	66.2	66.1	43.0	80.7

The original CCI showed the highest mean and median values for all estimates, both at national level and for the poorest and wealthiest quintiles (Table 2). At national level, average coverage with the revised CCI was 5.7 percentage points lower than for the original formulation, which is explained by the fact that DFPSmo, ANC4 and ORS had lower average coverage than the three indicators that they replaced, respectively DFPS, ANC1 and ORT. Mean coverage with the revised plus early initiation of breastfeeding CCI was almost six percentage points lower, because early initiation tended to be markedly lower than coverage with most of the other indicators in the formula. Addition of another low-coverage indicator–postnatal care for babies–further reduced the average level of the CCI.

The most comprehensive formulation (revised CCI plus early BF initiation plus tetanus vaccine plus postnatal care) showed the lowest national average values. When the analyses were restricted to the poorest or richest quintile, similar patterns were observed.

The correlations among the CCI formulations are shown in Table 3. As expected, all coefficients were positive and very high, ranging from 0.93 for the correlation between original CCI and the revised CCI plus early BF initiation to 0.99 for the correlation between the revised CCI plus early breastfeeding initiation plus tetanus toxoid and the revised CCI plus early breastfeeding initiation plus postnatal care for babies.

**Table 3:** Correlation matrix for the different formulations of the CCI in 47 countries.

	Original CCI	Revised CCI	Revised CCI + early BF initiation	Revised CCI + early BF initiation + post-natal care for babies	Revised CCI + early BF initiation + tetanus vaccine
Original CCI	1				
				
Revised CCI	0.97	1			
				
Revised CCI + early BF initiation	0.93	0.97	1		
				
Revised CCI + early BF initiation + post-natal care for babies	0.94	0.97	0.96	1	
				
Revised CCI + early BF initiation + tetanus vaccine	0.94	0.98	0.98	0.97	1
				
Revised CCI + early BF initiation + tetanus vaccine + post-natal care for babies	0.95	0.98	0.96	0.99	0.99
				

CCI = composite coverage index; BF = breastfeeding; all correlations with P<0.001.

**Fig 2:**
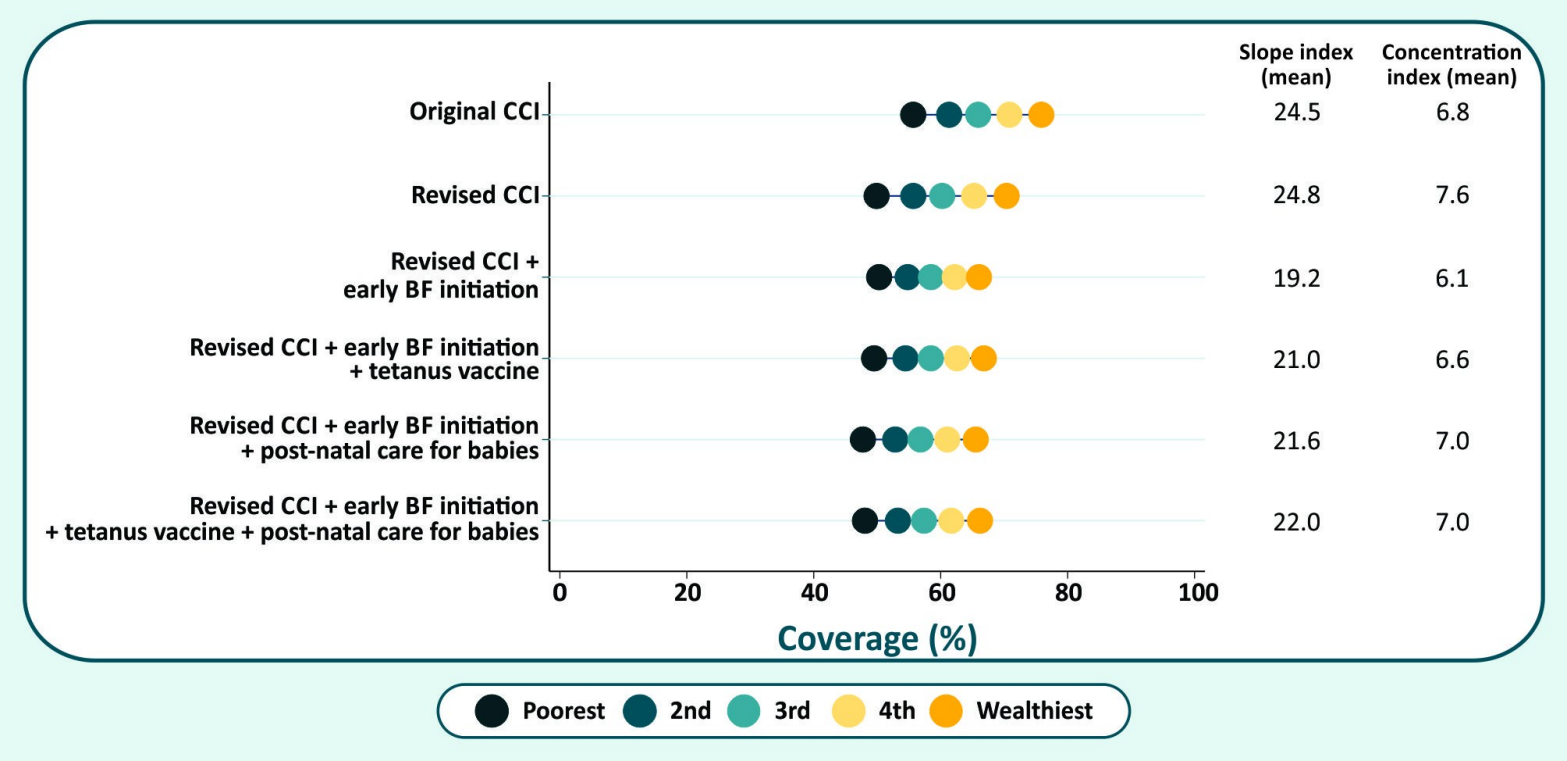
Average values by wealth quintile of the different formulations of the CCI, in 47 countries.

Absolute inequalities are shown graphically in Fig 2, an “equiplot” where each dot represents one wealth quintile. The widest inequalities observed for the revised CCI, and the narrowest inequalities for the revised CCI + early BF initiation formulation. Nevertheless, the magnitude of inequalities did not vary markedly from one formulation to another, with average levels ranging from 19.2 to 24.8 for the slope index, and 6.1 to 7.6 for the concentration index. Confidence intervals and ranges for the inequality measures are available in S2 Table.

**Fig 3:**
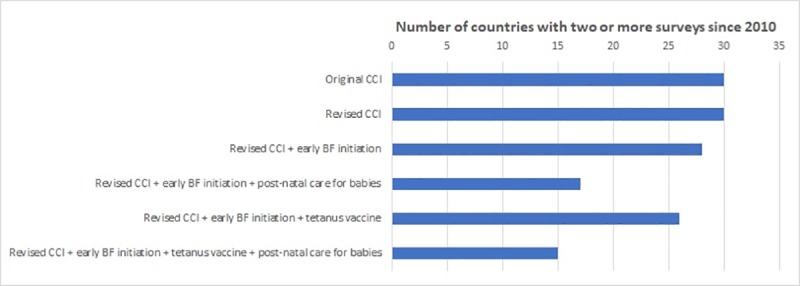
Information availability for time trend analysis for each CCI formulation.

Reporting on time trends is important for monitoring country progress, and we assessed the availability of data for such analyses. Thirty of the 60 countries with at least one measure of CCI available have had at least two surveys since 2010 in which the original and revised CCI could be calculated (Fig 3). The number of countries with more than one survey available is reduced as additional indicators are added to the CCI, particularly postnatal care for which information failed to be collected in some of the older surveys (Fig 3).

## Discussion

Monitoring progress in terms of the SDG goal 3.8 on universal health coverage has become a cornerstone of the health agenda for 2030. A list of 11 tracer coverage indicators has been proposed by WHO [15] which includes four indicators that are also part of the CCI: family planning, skilled birth attendance, measles vaccine and child pneumonia care. The UHC index also includes another seven tracer indicators (basic sanitation, malaria prevention, HIV treatment, TB effective treatment, tobacco control measures, hypertension treatment and diabetes treatment), of which sanitation and smoking among women are also collected in RMNCH surveys. However, the remaining five indicators will have to be derived from routine statistics or surveys of the adult population, which are currently not nearly as available or standardized as is the case for RMNCH surveys, which took place in over 100 countries since 2010. In addition, few LMICs will have sufficient data on these five indicators–except possibly for HIV treatment–to calculate time trends. For these reasons, monitoring coverage with essential RMNCH interventions is likely to remain the primary indicator for tracking universal health coverage and inequalities in most countries in the near future. Summary measures such as the CCI that combine information on RMNCH indicators along the continuum of care will play a central role in monitoring progress at national level, and in identifying subgroups of women and children who are being left behind. Summary indices are more precise in statistical terms than standalone coverage indicators, and are thus better suited for identifying overall trends and inequalities.

The original version of the CCI, based on the “coverage gap” that was proposed in 2008, was used in several analyses carried out by the Countdown initiative up to 2015 [16], when the revised version was introduced [7], as described above. Our present analyses show that the revised CCI resulted in national coverage estimates that were about five percent points lower compared to its original formulation. The reduction was larger among the poor than for the rich, as the more stringent coverage indicators included in the new version had lower coverage among the former. The addition of newborn indicators in the revised CCI did not change overall national coverage levels, but the value of the CCI decreased markedly in the richest quintiles. This was primarily due to low coverage with early breastfeeding initiation among the rich, compared to the coverage of other interventions (Fig 1). As a result, inclusion of newborn indicators led to a reduction in socioeconomic inequalities compared to the revised CCI, especially in terms of absolute inequalities. The addition of newborn care indicators other than early breastfeeding initiation reduced the number of surveys with available information, particularly for time trend analyses that require data from older surveys.

We used two criteria for identifying the optimal version of the CCI to be used in the near future. The first was data availability, in terms of the number of countries with recent and past information which are needed for time trend analyses. The second was the ability of the index to unveil socioeconomic inequalities. Based on these criteria, the revised CCI formulation appears to be the most appropriate. The addition of early initiation of breastfeeding led to a slight reduction in the number of countries with available data, but to an important reduction in the magnitude of inequalities, as this practice is unrelated to socioeconomic position in most countries as shown in earlier analyses [17]. This finding suggests that health workers are failing to promote earlier initiation, even for women from wealthy families who show high coverage with antenatal and delivery care.

Post-natal care shows a strong socioeconomic gradient even though average coverage is low, but data availability is limited. Still, there are differences in the way post-natal care is collected in DHS and MICS surveys, which could undermine between countries comparisons [18]. Additionally, recall bias could be present, especially in institutional births, as care might be provided in the absence of the mother. Also, the socioeconomic gradient in this variable could be driven by disparities in institutional delivery. Lastly, as mentioned, the revised version of the CCI already includes two indicators related to newborn health, namely antenatal and delivery care.

Other indicators included in the CCI, such as antenatal and delivery care, have their own limitations. ANC4 and SBA measure contact, rather than quality of care. For ANC4, several authors are proposing improvements such as accounting for timing of first visit, collection of blood and urine samples, and measurement of blood pressure [19–22]. Likewise, information on SBA does not take quality of care into account and this is not available in DHS or MICS surveys. This also applies to other components of the CCI; for example, immunization does not take the cold chain into account, nor does pneumonia careseeking tell us about whether the right drugs were provided. Yet, the original CCI was developed and used to monitor general progress in contact and utilization of RMNCH services and interventions [3], and there are at least two reasons for adhering to this approach. Firstly, these are standard global health indicators, adopted by international organizations. Secondly, the number of countries with available data on quality is restricted, and therefore one cannot calculate the CCI for countries with no information. As concern with quality of care increases [23], it is likely that available data will increase and the CCI may be revised in the future to incorporate this important dimension.

As a composite summary indicator, the CCI per se does not provide information on which interventions are lagging behind in terms of coverage, but this information can be readily obtained by policy makers and managers at national level. Its main advantage is to allow comparisons among countries in terms of levels and trends over time in contact with essential services and interventions.

## Conclusions

Our conclusion is that, although the addition of other newborn coverage indicators would be desirable for advocacy purposes, the disadvantages of adding such indicators outweigh the potential advantages. The revised version of the CCI is statistically robust, highly correlated to mortality and undernutrition, and able to document levels and trends in socioeconomic inequalities, and for these reasons we recommend is continued use as a comprehensive measure of coverage among the RMNCH continuum of care.

## Supporting information

S1 Table Surveys included and the coverage, slope index of inequality and concentration index for each of the different composite coverage index formulations. (XLSX)

S2 Table Inequality measures for the different indicators included and for CCI formulations in 47 countries. (XLSX)

## Abbreviations

ANC1: at least one antenatal care visit with skilled provider
ANC4: at least four antenatal care visits
SBA: skilled birth attendant
BCG: bacile Calmette_Guérin vaccine
CAREP: care-seeking for suspected childhood pneumonia
CCI: composite coverage index
DFPS: demand for family planning satisfied with any method
DFPSmo: demand for family planning satisfied with modern methods
DHS: Demographic and Health Survey
DPT: diphteria-pertussis-tetanus vaccine
EIBF: early breastfeeding initiation
LMIC: low- and middle-income countries
MICS: Multiple Indicators Cluster Survey
MSL: measles vaccine
ORS: oral rehydration salts for diarrhoea
ORT: oral rehydration therapy for diarrhoea
PNCALL: post-natal care visit by the baby within 48 hours after delivery
RMNCH: reproductive, maternal, newborn and child health
SDG: Sustainable Development Goals
TET: tetanus toxoid given during pregnancy
UHC: universal health coverage
